# Regional Odontodysplasia With Calcifying Hyperplastic Dental Follicle and Enameloid Conglomerates: Ground Section and Polarized Light Microscopic Findings

**DOI:** 10.7759/cureus.116692

**Published:** 2026-09-21

**Authors:** Vaishnavi N Setloor, Sahana Srinath, Shwetha Natraj, Ramya TR, Aryan Wadehra, Bhagyashree TK

**Affiliations:** 1 Department of Oral Pathology and Microbiology, Government Dental College and Research Institute, Bengaluru, IND; 2 Department of Paediatric Dentistry, Government Dental College and Research Institute, Bengaluru, IND

**Keywords:** enameloid conglomerates, ghost teeth, ground section, polarized light microscopy, regional odontodysplasia

## Abstract

Regional odontodysplasia (ROD) is a rare, non-hereditary developmental dental anomaly affecting enamel, dentin, pulp, and cementum within a localized segment of the dentition. Despite its striking clinical and radiographic presentation, detailed histopathologic characterization using ground sections and polarized light microscopy remains limited, and the associated soft-tissue pathology is poorly documented. We report two cases of ROD with contrasting clinical patterns, both evaluated using a combination of ground section, polarized light microscopy, and decalcified histopathologic analysis. The first case was an eight-year-old girl with recurrent pain and purulent discharge affecting the left maxilla and mandible simultaneously. The second was a 14-year-old girl with a hyperplastic gingival swelling and purulent discharge in the left mandibular anterior segment.

Ground sections in both cases demonstrated thin, hypoplastic enamel, an irregular dentino-enamel junction (DEJ) lacking its normal scalloped configuration, extensive interglobular dentin, and widened predentin zones. Polarized light microscopy revealed patchy, heterogeneous birefringence with a loss of the uniform pattern characteristic of normally mineralized dentin in both cases, and foci of enameloid aggregates were identified within the dentinal matrix. The second case additionally demonstrated an inflamed, hyperplastic dental follicle harboring proliferating odontogenic epithelial rests and multiple foci of whorled basophilic calcifications, findings consistent with a calcifying hyperplastic dental follicle (CHDF).

These cases expand the histopathologic spectrum of ROD and establish the diagnostic value of a combined assessment of hard and soft tissues. The presence of a CHDF in ROD involving unerupted permanent teeth is an underrecognized but important finding that may radiographically simulate an odontogenic cyst or neoplasm, underscoring the importance of submitting associated soft tissue for histopathologic evaluation when enlarged pericoronal tissue is present.

## Introduction

Regional odontodysplasia (ROD) is a rare, non-hereditary developmental anomaly affecting all dental tissues, including enamel, dentin, pulp, and cementum, within a localized segment of the dentition [1]. First described as “arrested tooth development” by Hitchin in 1934 and subsequently designated as odontodysplasia by Zegarelli et al. in 1963, the condition is characterized by simultaneous dysplastic involvement of tissues derived from both the ectoderm and the ectomesenchyme, which distinguishes it from isolated developmental defects affecting enamel or dentin. Clinically, affected teeth are malformed, hypoplastic, and yellowish-brown in color; radiographically, they display the pathognomonic “ghost-like” appearance, a consequence of markedly reduced mineralization and thinning of enamel and dentin, with enlarged pulp chambers, short, open-apexed roots, and enlarged pericoronal follicular spaces [2].

ROD predominantly affects children and adolescents, with a reported female predominance and a predilection for the maxillary dentition [3]. Most cases involve a single jaw quadrant, accounting for approximately 74.5% of documented cases; involvement extending beyond a single quadrant, including ipsilateral, contralateral, or bilateral patterns, is collectively reported in fewer than 20% of cases, with simultaneous ipsilateral bimaxillary involvement representing one of the rarest distributions [4]. The etiology remains unresolved, with proposed mechanisms encompassing localized vascular disturbance, somatic mutation, neural crest cell defects, viral infection, trauma, and ischemia during critical periods of odontogenesis [5].

While the radiographic features of ROD are well established, detailed tissue-level characterization remains sparse. A 2022 systematic review of 180 reported cases found that detailed histopathologic data were presented in fewer than half, underscoring the limited histopathologic characterization of this condition in the published literature [6]. The associated dental follicle, which is frequently enlarged in ROD and may harbor calcifying structures termed enameloid conglomerates or calcifying whorled nodules (CWNs), has likewise received limited systematic attention despite its diagnostic significance [7].

We report two cases of ROD with contrasting clinical patterns, both evaluated using a combination of ground section, polarized light microscopy, and decalcified histopathologic analysis. These techniques are complementary: ground sections preserve the mineralized architecture that decalcification destroys, polarized light demonstrates regional variation in the organization of the mineralized matrix, and decalcified sections permit assessment of the pulp, follicle, and associated soft tissue. The first case represents an uncommon instance of simultaneous ipsilateral bimaxillary involvement in the mixed dentition, affecting both primary teeth and the permanent maxillary first molar, with longitudinal radiographic documentation over five years. The second case involves an adolescent with unilateral mandibular involvement in whom histopathologic analysis of the associated dental follicle revealed a calcifying hyperplastic dental follicle (CHDF). Together, the cases document the range of distributions that ROD may exhibit and draw attention to follicular pathology that may be missed when soft tissue is not submitted for evaluation.

## Case presentation

Case 1

An eight-year-old girl presented with a history provided by her guardian. The guardian reported recurrent episodes of pain and swelling involving the left side of the upper and lower jaws since early childhood, with intermittent pus discharge.

History and Clinical Examination

The patient had been apparently normal at birth and remained asymptomatic until approximately three years of age, when pain and swelling had been first noted in the left posterior regions of both the maxilla and the mandible. Subsequent episodes had been recurrent and associated with tooth mobility and pus discharge. Over time, failure of eruption of teeth in the affected region had been observed, alongside progressive morphological deformity of erupted teeth. The condition remained strictly localized to the left side throughout the clinical course. Past dental history included excavation and composite restorations in teeth 65, 74, and 75, access opening with pulp therapy in tooth 62, and extraction of the hypoplastic tooth 61 following an acute abscess. The medical history was unremarkable, and there was no history of trauma, systemic illness, prolonged drug exposure, or radiation exposure; family history was non-contributory.

General examination showed a moderately built, well-nourished child. Extraoral examination showed a symmetrical face with no swelling, sinus tract, scar, or cervical lymphadenopathy. Intraoral soft-tissue examination revealed a localized erythematous gingival swelling associated with tooth 63, along with a sinus opening and purulent exudate. Hard tissue examination showed mixed dentition. The teeth present were 16, 55, 54, 53, 52, 51, 63, 64, 65, 26, 36, 75, 74, 73, 72, 71, 81, 82, 83, 84, 85, and 46. Teeth 63, 64, 65, and 26 were hypoplastic, with irregular crown morphology and yellowish-brown discoloration. A composite restoration was present in tooth 74, and active caries was noted in tooth 75. The right maxillary and mandibular teeth appeared clinically unaffected (Figure 1).

**Figure 1:**
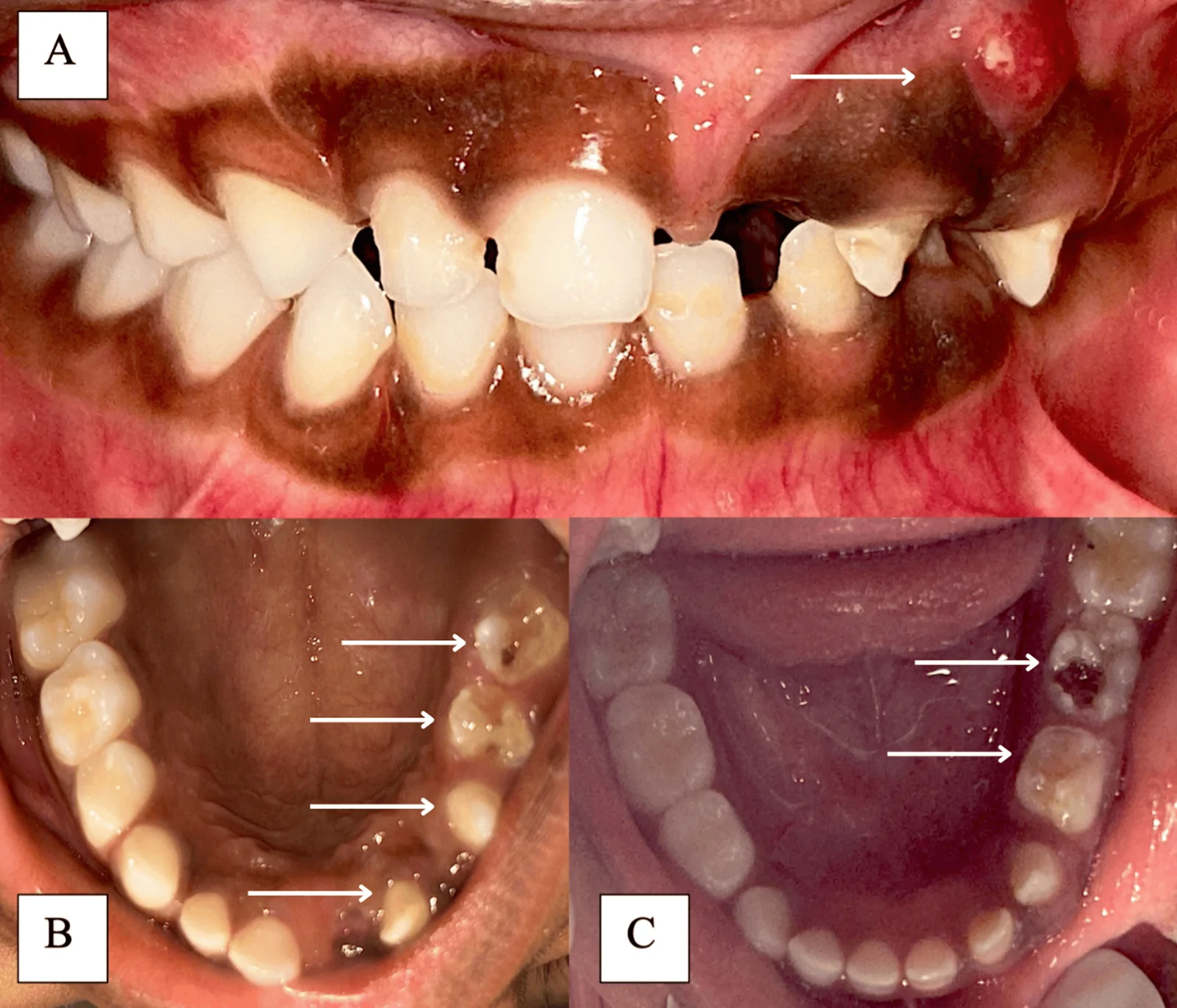
Case 1: intraoral clinical photographs (A) Frontal view; the arrow indicates the localized erythematous gingival swelling with a sinus opening and purulent discharge in relation to tooth 63. (B) Maxillary occlusal view; arrows indicate the hypoplastic crowns of teeth 63, 64, 65, and 26, showing irregular morphology and yellowish-brown discoloration. (C) Mandibular occlusal view; arrows indicate the composite restoration in tooth 74 and the active caries in tooth 75

Radiographic Examination

Serial panoramic radiographs obtained at three, six, and eight years of age, supplemented by intraoral periapical radiographs at the most recent visit, documented the affected teeth at successive stages of dental development. At three years of age, developing tooth buds in the left maxillary posterior segment had appeared ill-defined, irregular, and poorly organized, with markedly reduced radiopacity and loss of enamel-dentin distinction, producing an early ghost-tooth appearance. The right maxillary segment had shown normal morphology and radiodensity at this stage. At six years, the affected left maxillary posterior teeth had demonstrated a fully established ghost-tooth appearance with thin enamel and dentin, large pulp chambers, short roots with open apices, and delayed eruption of permanent successors. By eight years, these changes were more clearly established. Teeth 71-75 demonstrated reduced radiodensity compared with the contralateral teeth 81-85, together with enlarged pulp chambers, confirming ipsilateral mandibular involvement (Figure 2).

**Figure 2:**
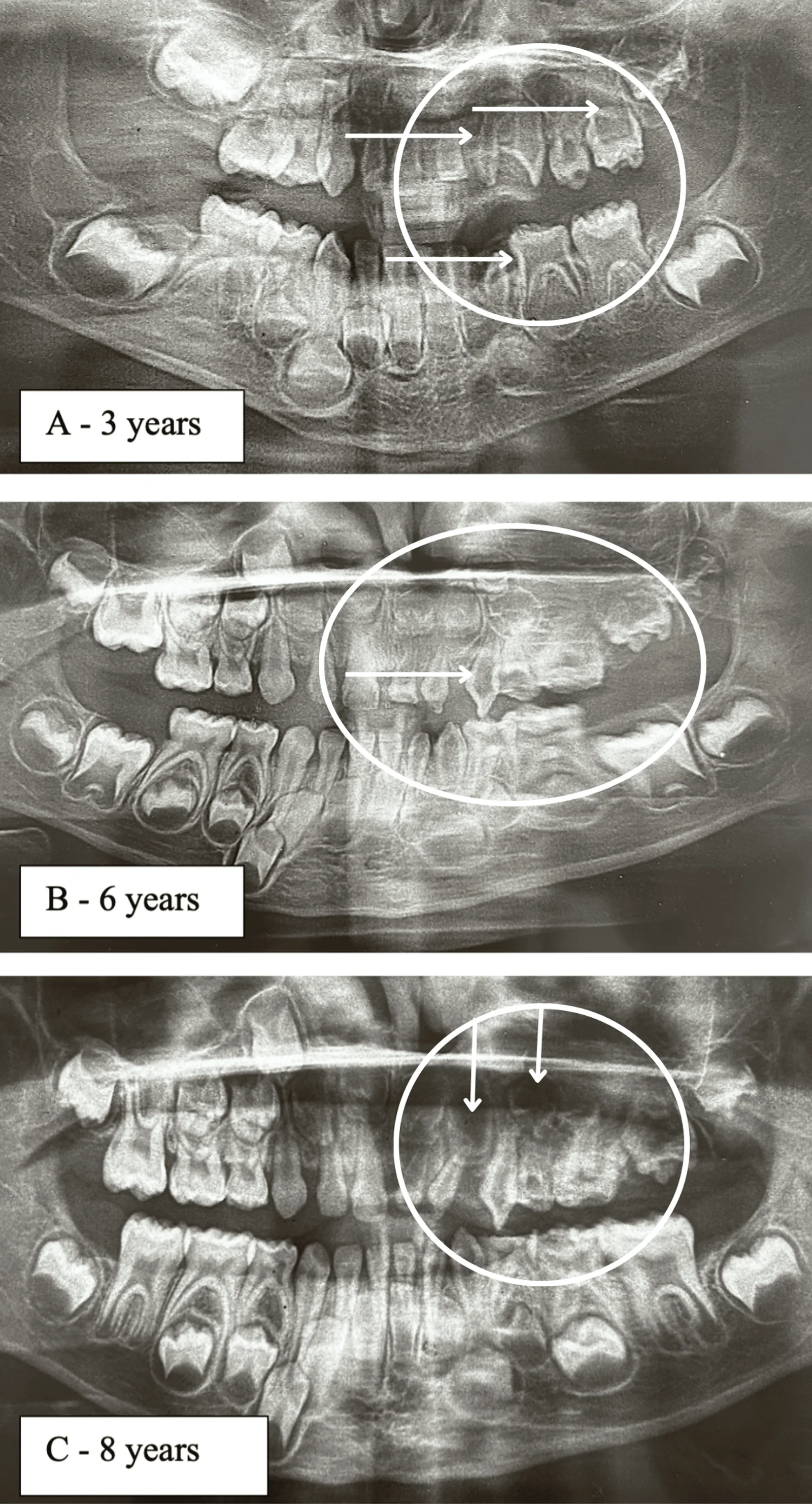
Case 1: serial panoramic radiographs over five years (A) At three years; arrows indicate the ill-defined, irregular, poorly organized developing tooth buds of the left maxillary posterior segment with markedly reduced radiopacity and loss of enamel-dentin distinction, with the left mandibular segment exhibiting wide pulp chambers. (B) At six years; arrow indicates the fully established ghost-tooth appearance of the affected left maxillary teeth, with thin enamel and dentin, enlarged pulp chambers, and short roots with open apices. (C) At eight years; arrows indicate the reduced radiodensity and enlarged pulp chambers of the affected left maxillary teeth and the hazy appearance of the tooth follicles

Histopathologic Examination

Tooth 64 was subsequently extracted following an acute abscess. Both extracted primary teeth, teeth 61 and 64, were submitted for combined ground section and decalcified histopathologic evaluation. Gross examination of tooth 64 revealed an irregularly shaped specimen measuring 1.2 × 0.9 × 1.0 cm, with a distorted, hypoplastic crown lacking normal anatomical contours, appearing pale yellowish-white, opaque, and chalky with a soft, friable consistency. The crown was reduced in dimension with surface irregularities and focal enamel loss; cusp anatomy was poorly defined with flattening and rounding of the occlusal surface. Roots were short, malformed, and incompletely developed with blunted open apices. Tooth 61 measured 0.8 × 0.5 × 0.8 cm and appeared small, malformed, and hypoplastic, with reduced incisal anatomy, pale yellowish-white coloration, and a soft, friable consistency. The root was short, slender, and incompletely developed with a blunted open apex (Figure 3).

**Figure 3:**
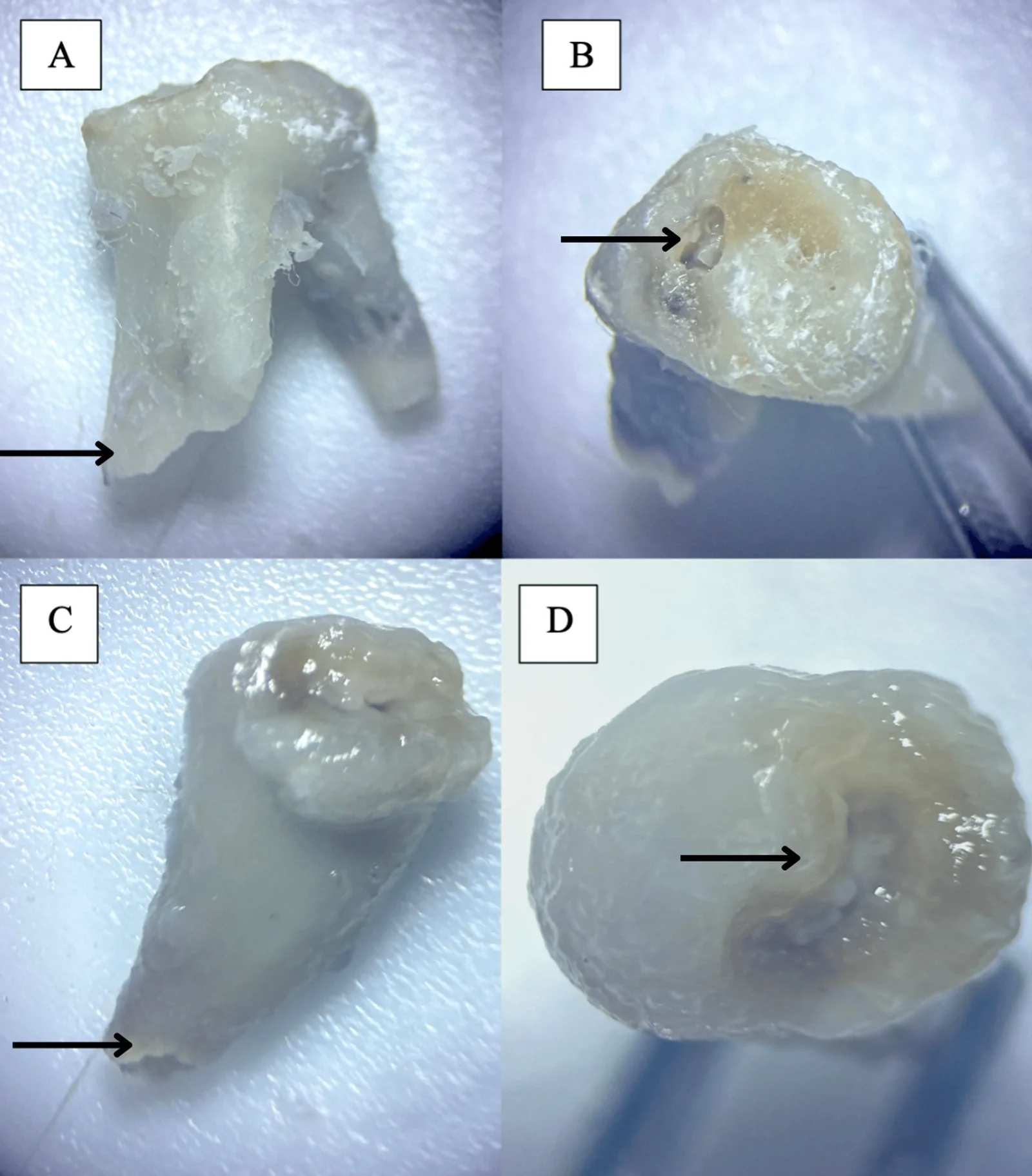
Case 1: gross examination of extracted primary teeth (A) Tooth 64; the arrow indicates the short, malformed, bifurcated roots with blunted open apices. (B) Tooth 64, occlusal view; the arrow indicates the central pulpal opening exposed by the markedly thin, friable hard-tissue walls. (C) Tooth 61; the arrow indicates the short, slender, incompletely developed root with a blunted open apex. (D) Tooth 61, occlusal view; the arrow indicates the prominent central hollow area

Ground section analysis demonstrated markedly thin, hypoplastic enamel with focal areas of discontinuity and regional absence. Enamel rods were poorly formed and disorganized, lacking normal prismatic architecture. The dentino-enamel junction (DEJ) was indistinct and entirely devoid of its normal scalloped contour. The dentin showed extensive interglobular areas: irregular, poorly fused globular zones representing defective mineralization fronts. Dentinal tubules were sparse, irregular, and disorganized, with amorphous, structureless areas. A widened predentin zone was evident along the pulpal aspect. The pulpal space was markedly enlarged, with focal calcifications. Cementum appeared thin and irregular but relatively better organized than the dentin, with visible cementocytes (Figure 4).

**Figure 4:**
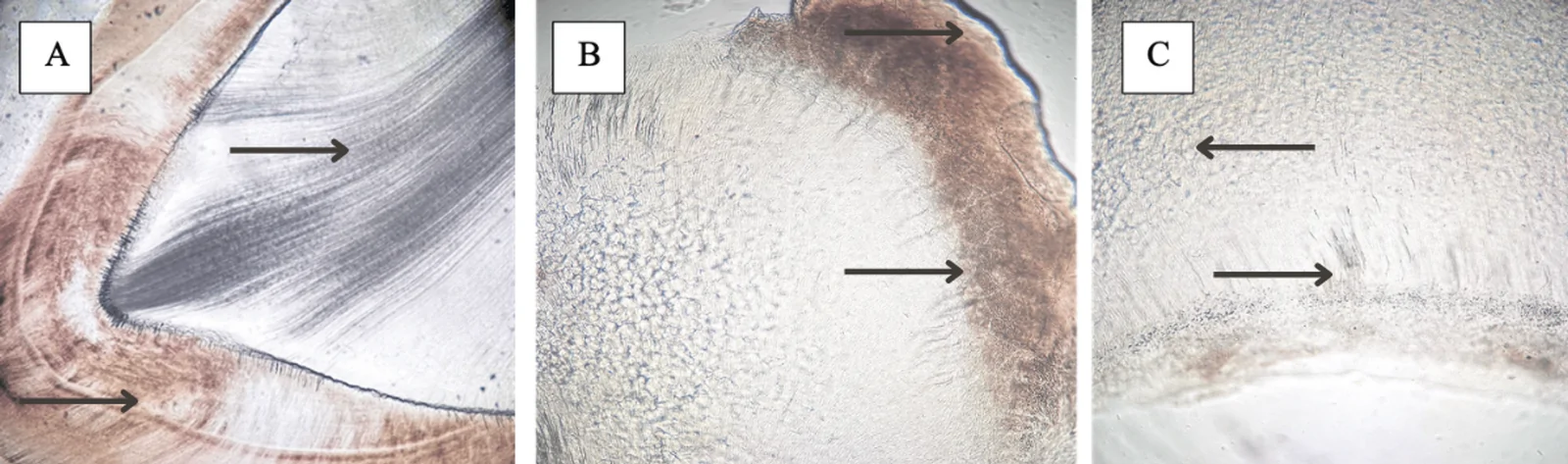
Case 1: ground sections of tooth 64 (unstained) (A) Overview of the section (×40); arrows indicate the enamel and the underlying dentin. (B) Higher-power view (×100); arrows indicate the markedly thin, hypoplastic enamel and the indistinct dentino-enamel junction, entirely lacking its normal scalloped configuration. (C) High-power view of the dentin (×200); arrows indicate the sparse, irregular, disorganized dentinal tubules and the amorphous, structureless interglobular zones

Polarized light microscopy demonstrated patchy, heterogeneous birefringence of the dentinal matrix. The dentin exhibited irregular blue-gray birefringence interspersed with non-birefringent dark zones corresponding to interglobular dentin, directly demarcating areas of defective mineralization. There was also a striking polychromatic birefringence pattern with coexisting orange-red and blue interference colors within adjacent dentinal zones, indicating marked variation in optical retardation between adjacent regions of the dentinal matrix. This polychromatic, non-uniform pattern contrasts fundamentally with the uniform pale blue-white birefringence of normally mineralized dentin (Figure 5) [8].

**Figure 5:**
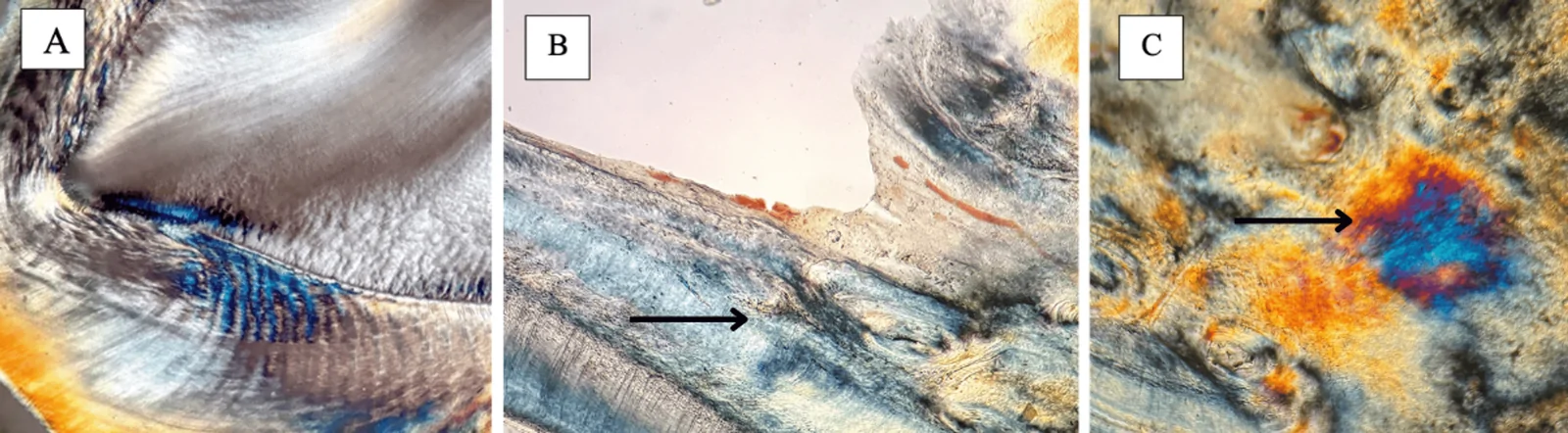
Case 1: ground sections viewed under polarized light (unstained) (A) Normal tooth for comparison (×100); the arrow indicates the uniform pale blue-white birefringence of normally mineralized dentin. (B) Affected dentin (×200); the arrow indicates the non-birefringent dark zones corresponding to interglobular dentin within the patchy blue-gray birefringent matrix. (C) Affected dentin (×200); the arrow indicates the polychromatic birefringence, with coexisting orange-red and blue interference colors in adjacent dentinal zones reflecting heterogeneous birefringence within the dentinal matrix

Decalcified hematoxylin and eosin-stained sections revealed thin and irregular enamel matrix remnants. Irregular globular enameloid conglomerates, which are basophilic deposits lacking normal prismatic architecture, were identified in focal areas. The dentin was poorly organized, with wavy, irregularly spaced dentinal tubules; interglobular areas and globular calcifications were evident throughout. Wide predentin zones were noted. The enlarged pulpal space contained multiple pulp stones, including true denticles with enclosed tubular structures and false denticles with lamellar calcified deposits, alongside areas of extravasated erythrocytes within the pulp stroma. Cementum, where identifiable, appeared thin and irregularly deposited (Figures 6, 7).

**Figure 6:**
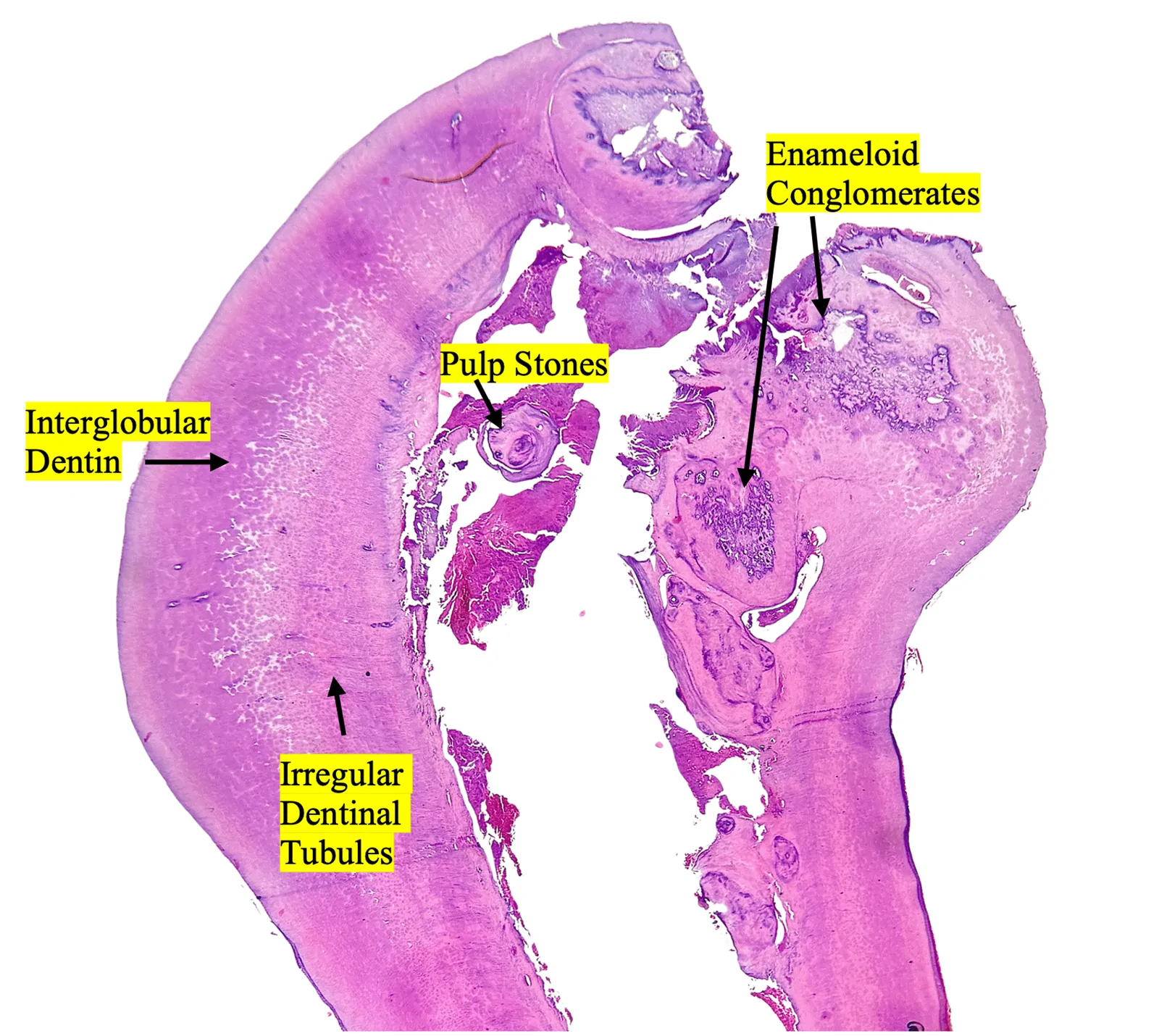
Case 1: decalcified section of tooth 61 Hematoxylin and eosin, ×100. Arrows indicate enameloid conglomerates: basophilic, irregular, globular calcified deposits lacking normal prismatic architecture, located within the dentinal matrix adjacent to the pulp chamber wall. Arrows also indicate the extensive interglobular dentin and irregular dentinal tubules, and the pulp stones within the enlarged pulp chamber

**Figure 7:**
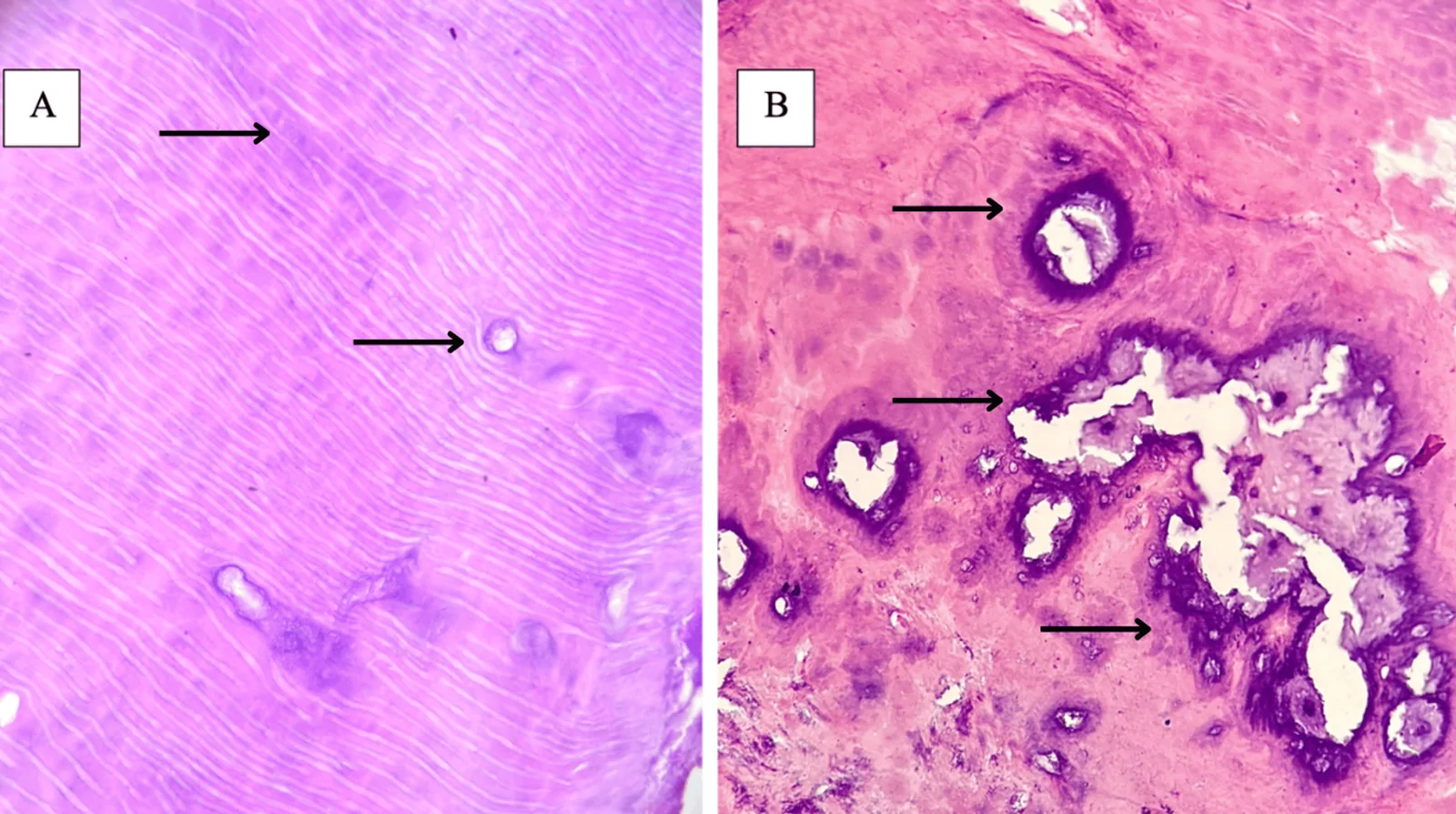
Case 1: decalcified sections (A) Dentinal matrix (hematoxylin and eosin, ×400); arrows indicate the wavy, irregularly spaced dentinal tubules and the interglobular dentin with globular calcifications. (B) Dentinal matrix (hematoxylin and eosin, ×400); arrows indicate the globular basophilic enameloid conglomerates

A confirmed diagnosis of ROD was established. Strip composite crowns were placed on teeth 65 and 75 to increase the vertical dimension (Figure 8).

**Figure 8:**
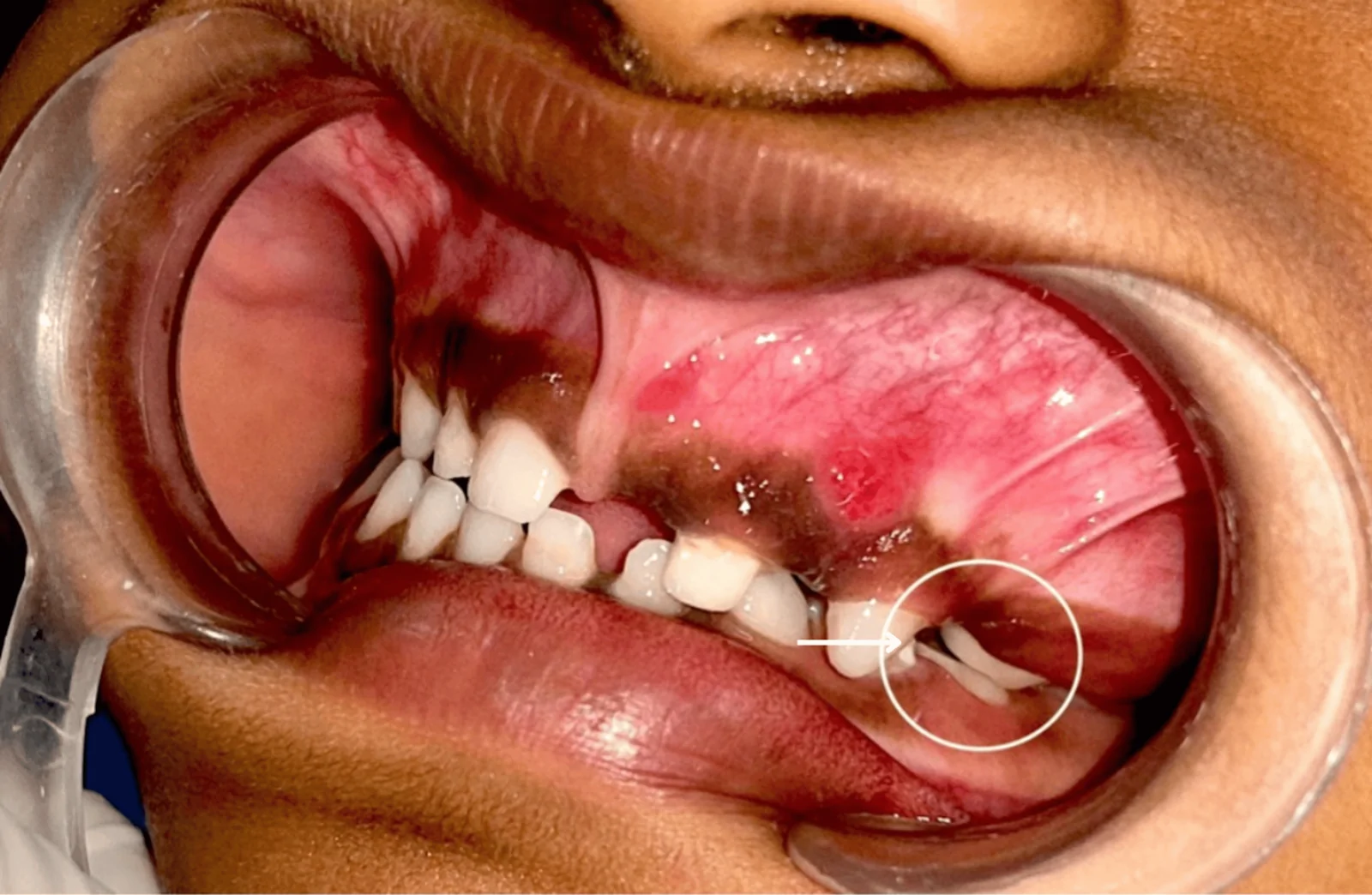
Case 1: post-treatment intraoral photograph The arrow and circled area indicate the strip composite crowns placed on teeth 65 and 75

Case 2

A 14-year-old girl presented with a chief complaint of progressive swelling in the left mandibular anterior and premolar region for one year and intermittent pus discharge from the lower anterior teeth for two years. The swelling had been initially small and had gradually increased in size. No history of trauma, systemic illness, prolonged medication, or radiation exposure was elicited; medical and family histories were non-contributory.

Clinical Examination

General examination was unremarkable. Extraoral examination revealed incompetent lips with no facial asymmetry, extraoral swelling, sinus tract, scar, or cervical lymphadenopathy. Intraoral soft-tissue examination revealed a solitary growth in the region of the left mandibular anterior and premolar segment, measuring approximately 4 cm × 1 cm in its greatest anteroposterior and buccolingual dimensions, roughly oval in shape, extending from the mid-coronal level of the premolar region to the mucogingival junction, and pale pink to erythematous in color. On palpation, the growth was firm to hard in consistency, non-tender, non-fluctuant, and non-pulsatile. Hard tissue examination revealed teeth 31, 32, 33, and 34 to be malformed, hypoplastic, and discolored. Tooth 35 was erupted in a lingual position. The right mandibular and bilateral maxillary dentitions appeared clinically unaffected. The clinical differential diagnosis for the gingival swelling included peripheral ossifying fibroma, peripheral giant cell granuloma, and fibrous hyperplasia, while the widened pericoronal radiolucency raised the possibility of a dentigerous cyst (Figure 9).

**Figure 9:**
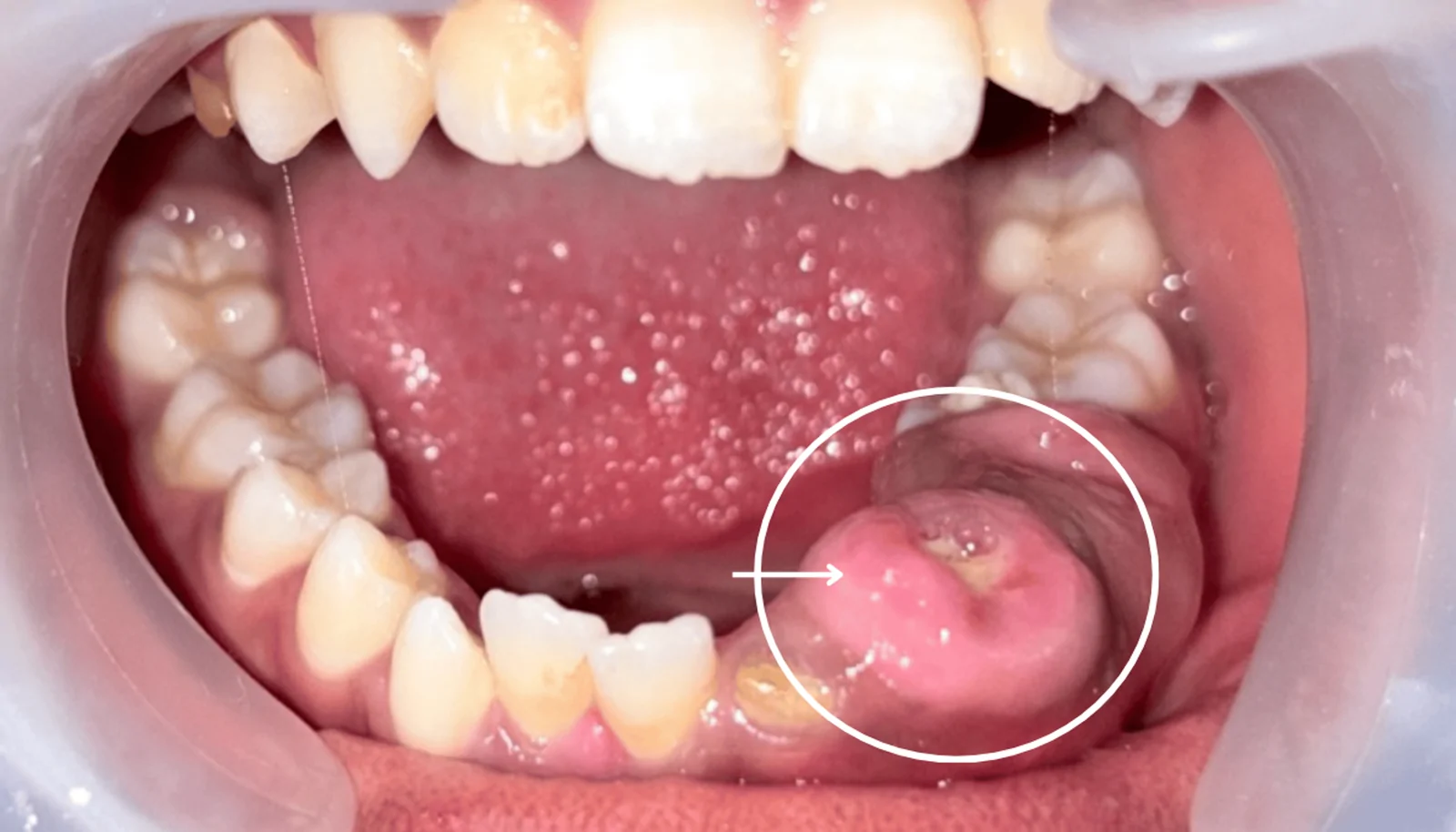
Case 2: intraoral examination The arrow indicates the well-defined lobulated hyperplastic soft-tissue growth in the left mandibular anterior to premolar segment, measuring approximately 4 × 1 cm and overlying the partially erupted teeth 31 and 32

Radiographic Examination

Orthopantomography and intraoral periapical radiographs of the affected region demonstrated the characteristic ghost-like radiographic appearance of teeth 31-35, with markedly reduced and homogeneous radiodensity, indistinct enamel-dentin differentiation, enlarged pulp spaces, and short roots with incompletely formed open apices. A widened pericoronal radiolucency was present around the crown of the partially erupted tooth 31, corresponding to the enlarged follicular tissue subsequently removed at incisional biopsy (Figure 10).

**Figure 10:**
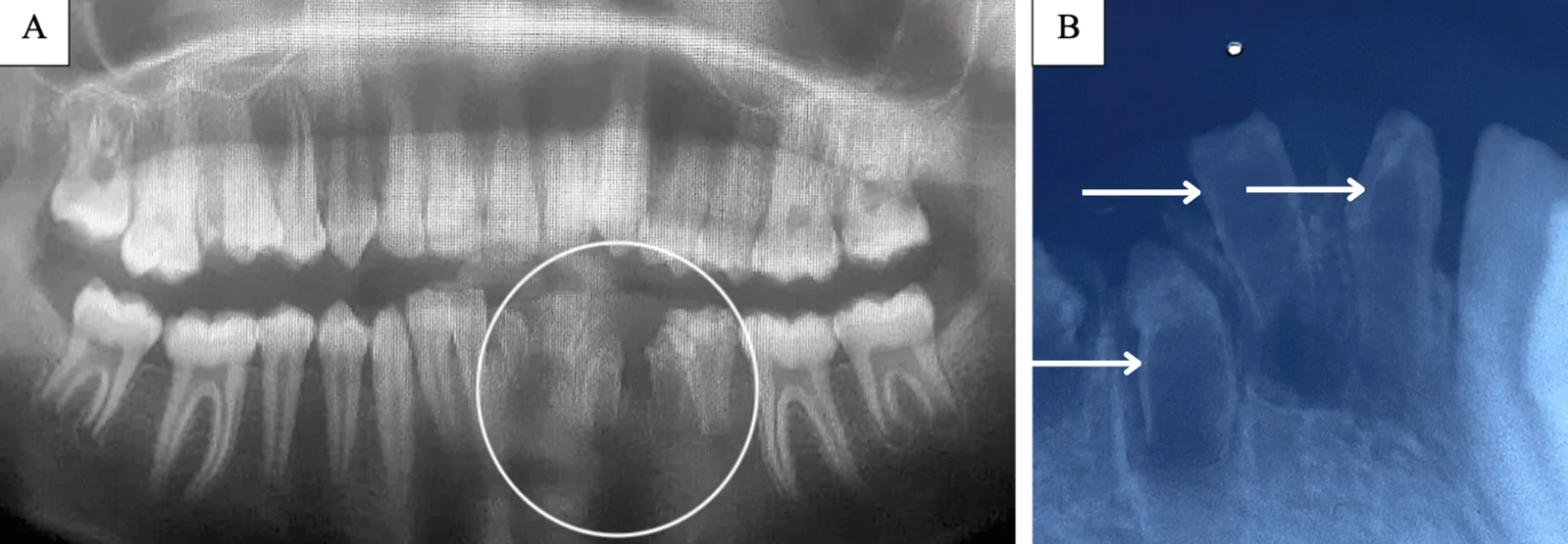
Case 2: radiographic findings (A) Orthopantomograph: the circled area indicates the ghost-like appearance of teeth 31-35 with markedly reduced homogeneous radiodensity, indistinct enamel-dentin differentiation, enlarged pulp spaces, short roots with open apices, and a widened pericoronal radiolucency around the crown of the partially erupted tooth 31. (B) Intraoral periapical radiograph; arrows indicate the indistinct hard-tissue outlines and markedly enlarged pulp spaces of the left mandibular anterior teeth

Histopathologic Examination

An incisional biopsy was performed. The specimen comprised six hard tissue fragments, the largest measuring 1.2 × 0.6 × 0.4 cm, irregular, rough, brownish, and hard, and one soft-tissue fragment measuring 3 × 1.4 × 0.4 cm, irregular, rough, whitish, and firm in consistency (Figure 11).

**Figure 11:**
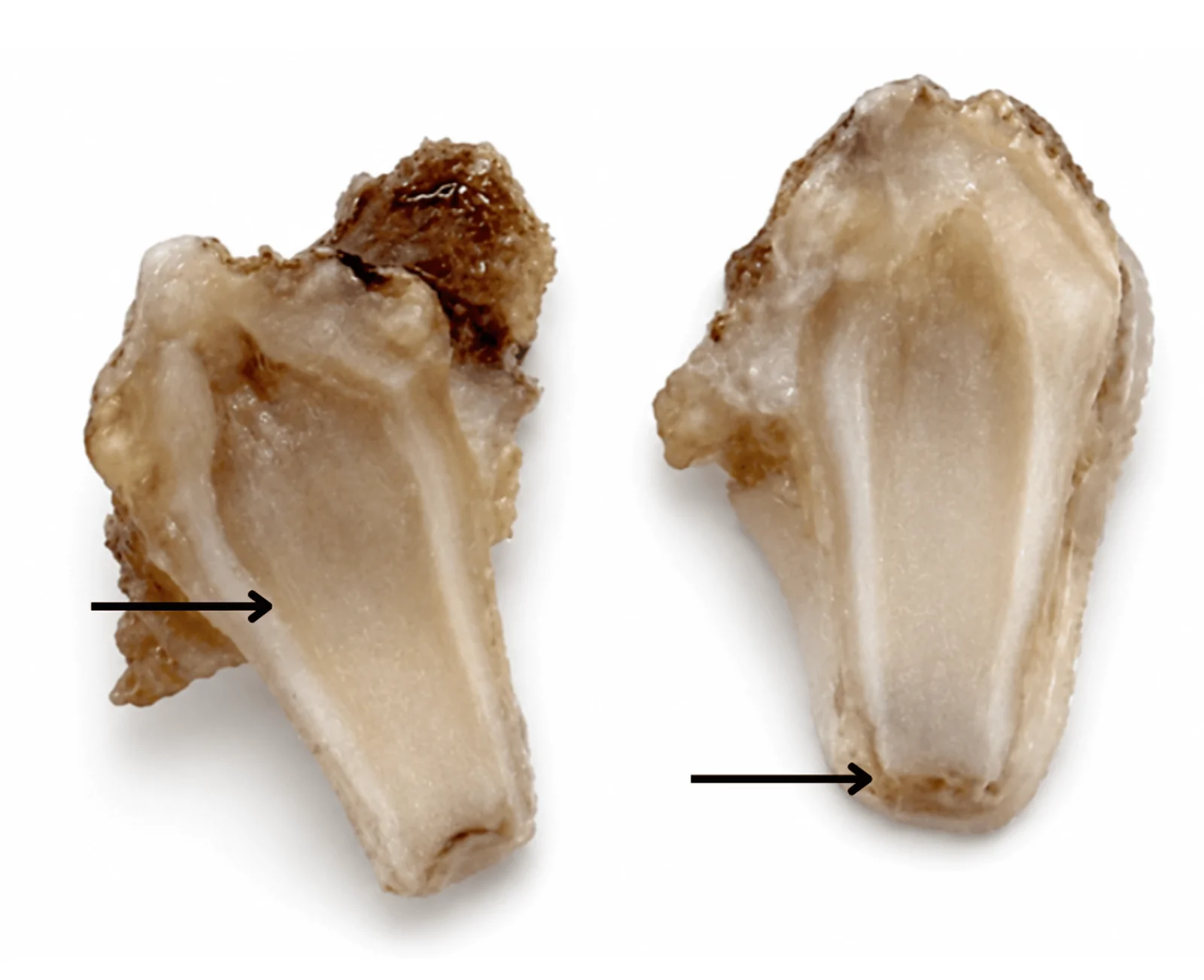
Case 2: gross examination of the biopsy specimen Tooth 31, sectioned longitudinally into two halves. Arrows indicate the markedly enlarged pulp chamber occupying the majority of the tooth volume and the short, incompletely formed root with a blunted, open apex

Ground section analysis of the hard tissue specimen revealed severely disrupted dental hard tissue architecture. Enamel appeared markedly thin and hypoplastic with focal discontinuities. The DEJ was indistinct, irregular, and entirely lacked the normal scalloped configuration. Enamel rods were poorly formed and disorganized. The dentin demonstrated extensive interglobular spaces with sparse, irregular, and disorganized dentinal tubules. Marked enlargement of the pulpal space was evident throughout (Figure 12).

**Figure 12:**
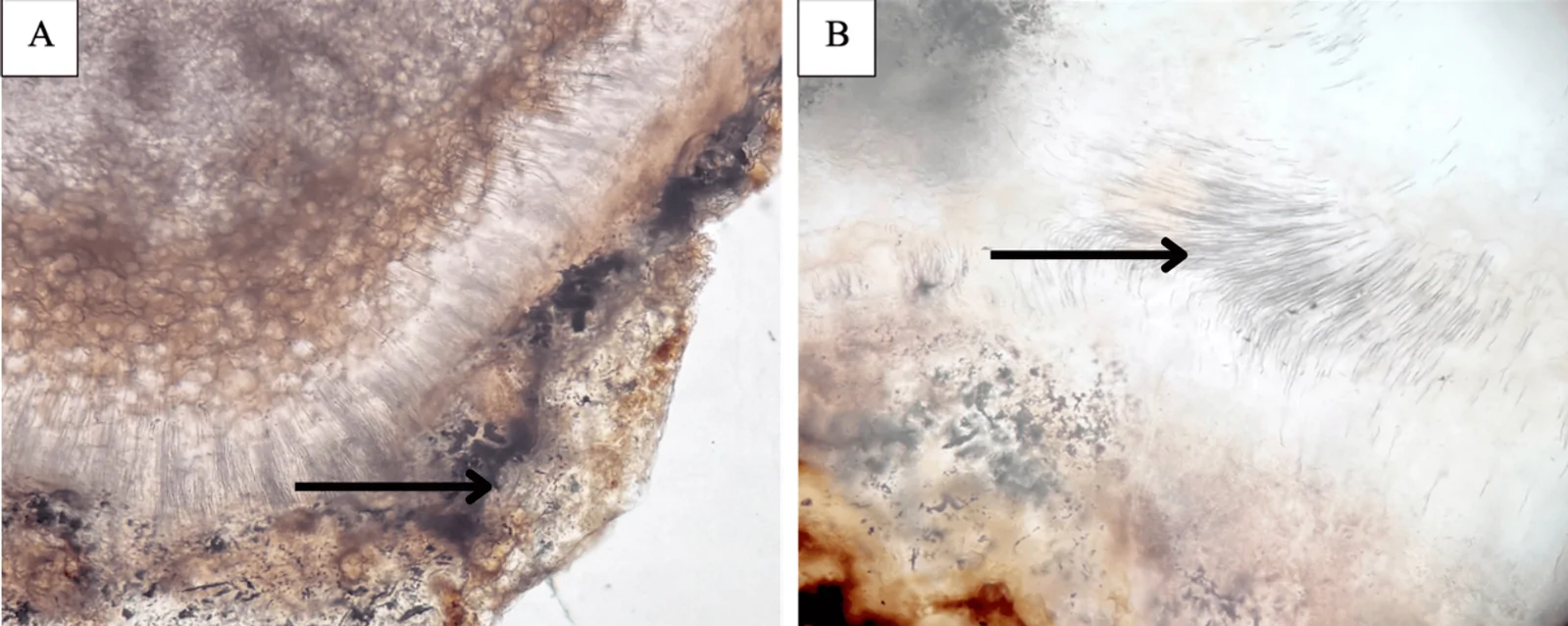
Case 2: ground sections of the hard tissue specimen (unstained) (A) Low-power view (×40); the arrow indicates the markedly thin, hypoplastic enamel and the irregular dentino-enamel junction lacking normal scalloping. (B) Higher-power view (×100); the arrow indicates the extensive interglobular spaces and the focal discontinuities in dentinal tubule arrangement

Polarized light microscopy demonstrated heterogeneous and patchy birefringence of the dentin. The dentinal matrix exhibited an abnormal orange-red birefringent zone indicating altered optical retardation relative to the surrounding matrix. The DEJ appeared as an intensely bright white zone under crossed polarizers, reflecting the abrupt structural transition at an irregular junction, while the interglobular dentin areas manifested as weakly birefringent or non-birefringent gray-white patches. This polychromatic, patchy pattern contrasts fundamentally with the uniform pale blue-white birefringence of normally mineralized dentin, indicating regional loss of the optical anisotropy that characterizes organized mineralized matrix (Figure 13).

**Figure 13:**
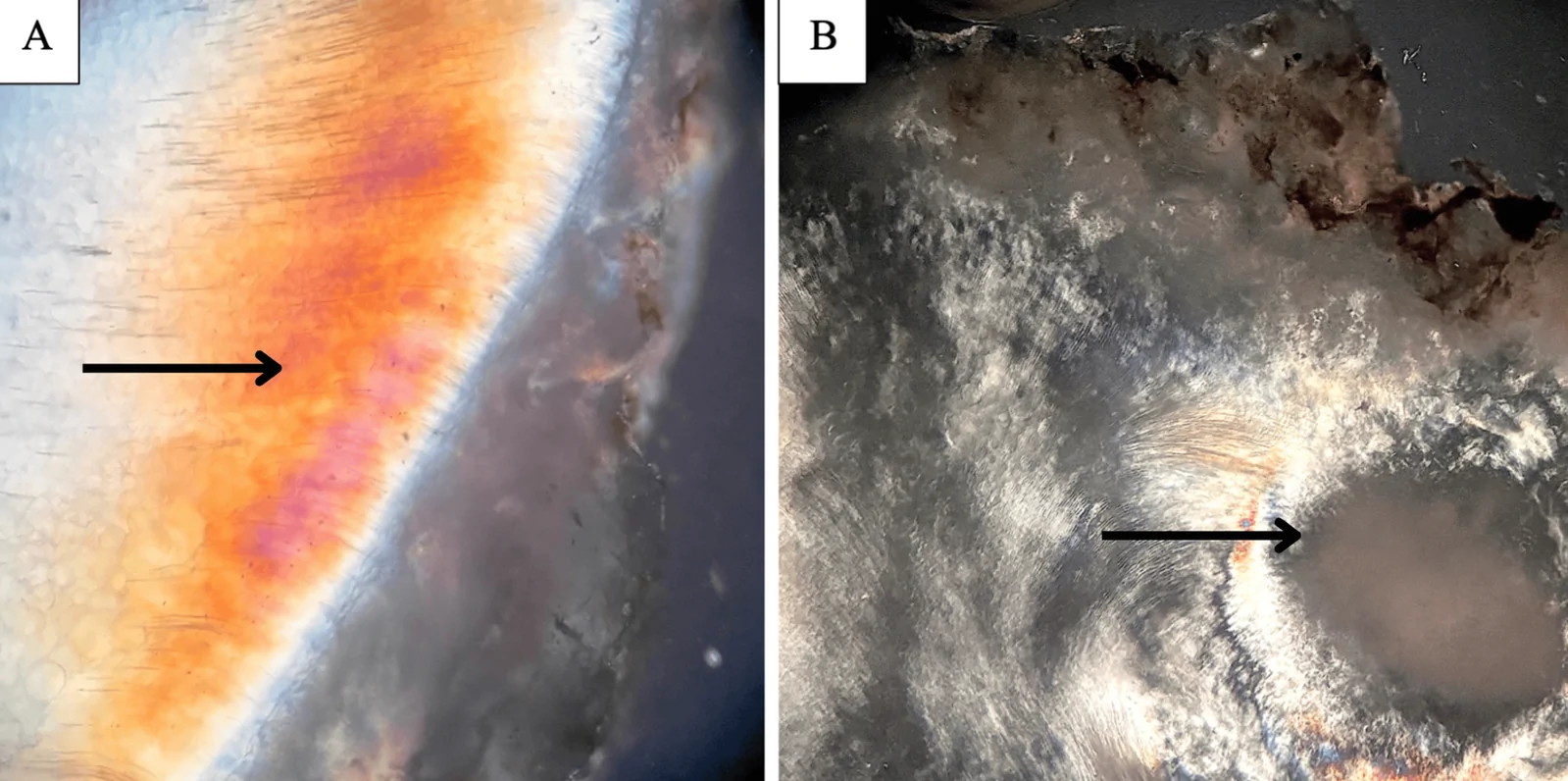
Case 2: ground sections viewed under polarized light (unstained) (A) Dentino-enamel junction (×200); the arrow indicates the abnormal orange-red birefringent zone within the dentin and the intensely bright white band at the junction. (B) Dentin (×200); the arrow indicates the large central non-birefringent dark zone representing defectively mineralized dentin lacking the organized structure that produces birefringence

Decalcified hematoxylin and eosin-stained sections of the hard tissue revealed thin and irregular enamel matrix remnants, poorly organized dentin with wide predentin zones, irregular tubular architecture, numerous interglobular areas, and focal enameloid aggregates within the dentinal matrix. The pulpal space was markedly enlarged with areas of extravasated erythrocytes. Cementum, where identifiable, appeared thin and irregularly deposited.

Histopathologic examination of the soft-tissue specimen revealed stratified squamous epithelium with elongated rete ridges, in areas ulcerated and replaced by a fibrinopurulent membrane. The underlying connective tissue was fibrocellular with endothelial-lined blood vessels. The fibrous stroma contained numerous proliferating odontogenic epithelial rests arranged in islands and cords, and multiple discrete foci of whorled basophilic calcifications, morphologically consistent with CWNs. A dense chronic inflammatory cell infiltrate was present, predominantly composed of plasma cells with occasional binucleated forms; scattered multinucleated giant cells and mast cells were also identified (Figure 14).

**Figure 14:**
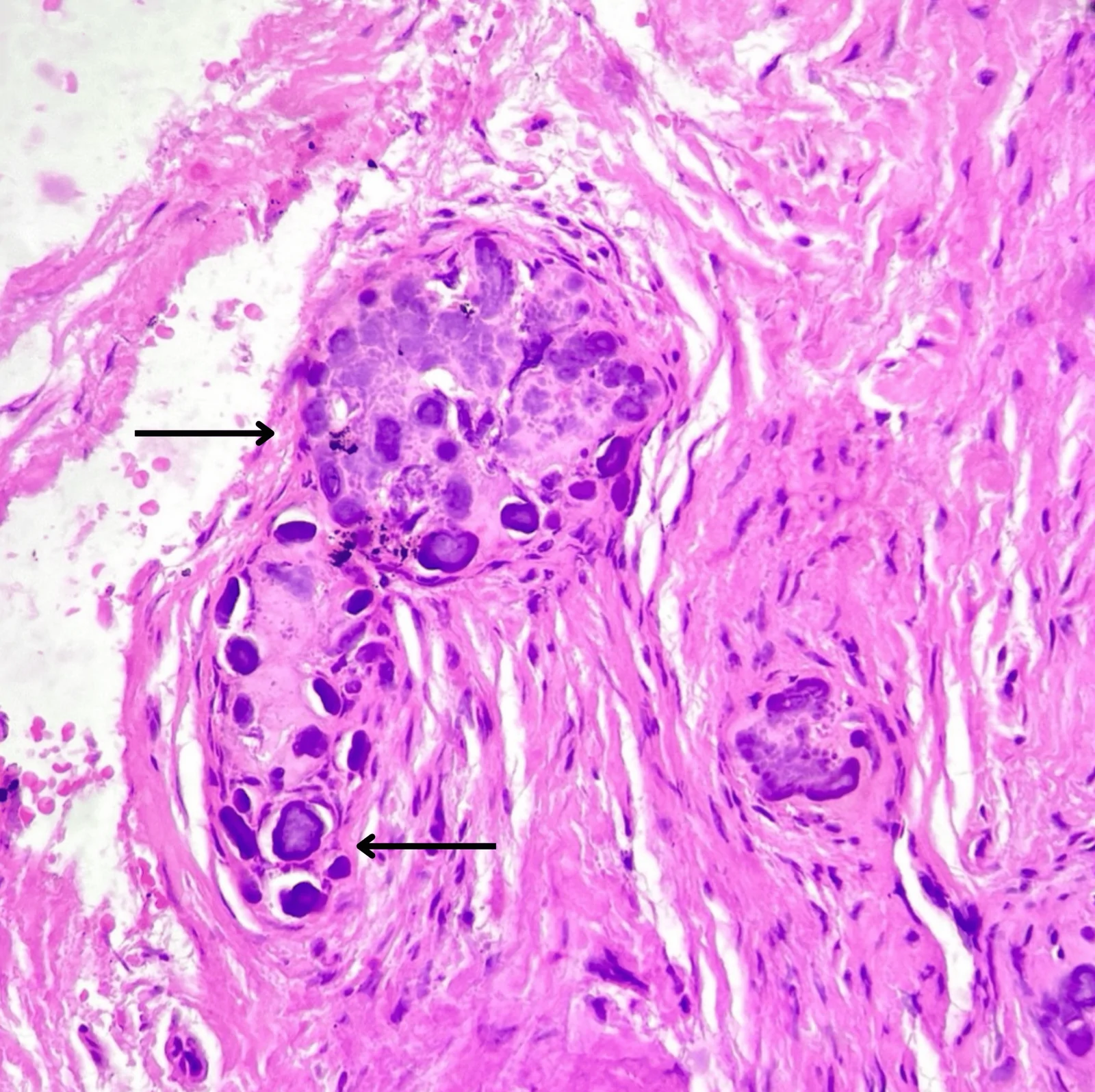
Case 2: decalcified section of the soft-tissue specimen Hematoxylin and eosin, ×400. Arrows indicate the whorled basophilic calcifications: calcifying whorled nodules enclosed within odontogenic epithelial islands in the fibrocellular connective tissue stroma

The histopathologic impression was ROD (ghost teeth) associated with an inflamed hyperplastic dental follicle with CWNs. The affected teeth were subsequently extracted, and the patient was referred for prosthetic and orthodontic rehabilitation. The patient was reviewed at six weeks postoperatively, with satisfactory healing of the extraction sites, and remains under active follow-up.

## Discussion

ROD is an uncommon developmental anomaly in which all dental hard tissues fail to form normally within a contiguous segment of the dentition, producing a clinical and histopathologic picture that is unlike any other dental developmental disorder. The two cases described here are individually notable and together illustrate the breadth of variation that ROD can manifest across age, dentition type, jaw distribution, and histopathologic complexity.

The simultaneous ipsilateral involvement of both the maxilla and the mandible in the younger patient places this presentation among an uncommon minority of reported cases, as a single jaw quadrant is affected in approximately 74.5% of documented cases [6]. This distribution is compatible with the proposed localized vascular disturbance hypothesis, which suggests that regional ischemia during odontogenesis may affect contiguous territories of both jaws [9]. The present cases cannot establish ischemia as the underlying mechanism, as no vascular assessment was performed and no cutaneous vascular abnormality was identified. Both patients shared the classical clinical triad of hypoplastic malformed teeth, recurrent periapical infection, and failure of normal eruption. The serial panoramic radiographs obtained at three, six, and eight years in the younger patient provide longitudinal documentation rarely described in published reports, with persistence of ghost-tooth changes over five years consistent with the non-progressive, non-resolving nature of ROD as a developmental anomaly.

Ground section histopathology

The ground section findings in both cases exhibited thin hypoplastic enamel, an irregular DEJ with loss of scalloping, extensive interglobular dentin, widened predentin zones, and pulpal enlargement [10]. The irregular DEJ deserves particular emphasis because its loss of scalloping has been interpreted as reflecting a failure of the reciprocal inductive signalling between the inner enamel epithelium and the dental papilla, a disruption that defines ROD as a disorder of odontogenesis itself and not merely a post-formative mineralization defect [11]. This is reinforced by the finding of enameloid aggregates in both cases: irregular globular basophilic deposits within the dentin lacking normal prismatic architecture, consistent with aberrant enamel matrix deposition attributed to defective ameloblast differentiation.

Polarized light microscopy

The polarized light microscopic findings in both cases provide a mechanistically informative dimension that is not apparent on routine ground sections alone. In normal dentin, the organized parallel orientation of hydroxyapatite crystals along collagen fibril axes produces uniform pale blue-white birefringence under crossed polarizers. In both cases, this uniform pattern was replaced by patchy, heterogeneous birefringence, with non-birefringent or weakly birefringent interglobular zones and a widened non-birefringent predentin band, indicating regional loss of the optical anisotropy that characterizes organized mineralized matrix. The second case additionally demonstrated an abnormal orange-red birefringent zone in the dentin, reflecting altered optical retardation relative to the surrounding dentinal matrix.

Calcifying hyperplastic dental follicle

A diagnostically significant finding of these cases is the histopathologic characterization of the dental follicle as an inflamed CHDF with CWNs, a recognized but underreported association of ROD in the permanent dentition that is frequently overlooked when soft tissue is not submitted alongside hard tissue specimens for histopathologic evaluation [12]. Enlargement of the pericoronal follicular tissue is a recognized feature of ROD involving unerupted teeth, attributed to anomalous inductive interactions between the dysplastic dental epithelium and the surrounding ectomesenchyme.

The CWNs identified in the second case were discrete basophilic, whorled, concentrically laminated calcified structures within the follicular stroma. These terms describe different features: "calcifying whorled nodule" refers to the characteristic architecture, in which stromal cells are arranged in a whorled or spiral configuration around foci of calcification, whereas "enameloid," "cementoid," and "psammomatoid" describe the nature of the calcified material, which may vary within and between nodules [13]. Such nodules are reported principally in multiple calcifying hyperplastic dental follicles and in regional odontodysplasia [14], and the follicular changes in the two conditions are histologically comparable, with ROD distinguished by the accompanying dental malformation [12].

The present case reinforces this association and highlights that the enlarged follicular radiolucency observed radiographically in ROD involving unerupted teeth may reflect an underlying CHDF, a finding with direct diagnostic implications, as it can simulate a dentigerous cyst or odontogenic neoplasm on imaging and will be missed entirely if soft tissue is not submitted for histopathologic evaluation.

Differential diagnosis

The principal differential diagnoses for ROD include amelogenesis imperfecta, dentinogenesis imperfecta, dentin dysplasia, Turner hypoplasia, and hypophosphatasia. Amelogenesis imperfecta affects only enamel, is typically generalized and hereditary, and does not involve dentin or pulp [15]. Dentinogenesis imperfecta demonstrates pulp obliteration rather than enlargement, bulbous crowns, and a hereditary pattern [16]. Dentin dysplasia presents with rootless or short-rooted teeth without the combined enamel defect or ghost-tooth radiographic appearance [17]. Turner hypoplasia affects single teeth secondary to periapical infection of primary predecessors, not contiguous segments [18]. Hypophosphatasia, which can produce hypomineralization and premature exfoliation, is a systemic metabolic disorder with characteristic skeletal and biochemical abnormalities absent in both cases [19].

Management

The histopathologic alterations described here account directly for the clinical behavior of the affected teeth. The markedly thin, hypoplastic enamel and the irregular DEJ lacking its normal scalloped configuration reduce the mechanical interlocking that normally resists shear at this interface, predisposing to enamel fracture and loss under function. The extensive interglobular dentin and sparse, disorganized dentinal tubules leave a hypomineralized, friable dentinal matrix that is readily penetrated by carious and bacterial invasion, while the markedly enlarged pulp chamber places the pulp in close proximity to the external surface. Together, these features explain the rapid progression from surface breakdown to pulpal necrosis and periapical abscess formation.

These same features govern treatment. The soft, friable hard tissue provides poor support for conventional restorations and unreliable bonding, since the disorganized enamel prisms and hypomineralized dentin compromise both etch patterns and hybrid layer formation. Short, open-apexed roots limit the prognosis of endodontic intervention and reduce retention for any restoration placed. The enlarged pericoronal follicular tissue, which in the second case harbored a calcifying hyperplastic dental follicle, contributes to failure of eruption and must be assessed histopathologically at the time of surgical removal rather than assumed to be an unremarkable follicle.

The management of ROD remains individualized and ranges from conservative monitoring of asymptomatic teeth to extraction with prosthetic or orthodontic rehabilitation, guided by symptom severity, infection risk, and the patient’s age. In the first case, teeth 61 and 64 were extracted following acute abscess formation and submitted for histopathologic evaluation, which established the diagnosis. The remaining affected teeth were managed conservatively, with strip composite crowns placed on teeth 65 and 75 to restore form and function. In the second case, extraction was selected over conservative management because of the extent of involvement, recurrent abscess formation, and the non-restorable structure of the affected teeth. The clinical, radiographic, and histopathologic features of both cases are compared in Table 1.

**Table 1:** Comparison of clinical, radiographic, and histopathologic features of the two cases Teeth are designated using FDI notation CHDF: calcifying hyperplastic dental follicle; CWN: calcifying whorled nodule; DEJ: dentino-enamel junction; FDI: Fédération Dentaire Internationale

Feature	Case 1	Case 2
Age at presentation	8 years	14 years
Sex	Female	Female
Dentition	Mixed dentition	Permanent dentition
Jaw and segment involved	Left maxilla and left mandible	Left mandible
Teeth affected	61, 63, 64, 65, 26	31, 32, 33, 34, 35
Presenting symptoms	Recurrent pain and swelling with intermittent pus discharge	Progressive gingival swelling with intermittent pus discharge
Duration of symptoms	Approximately 5 years	Swelling for 1 year; discharge for 2 years
Soft-tissue findings	Localized erythematous gingival swelling with sinus opening in relation to tooth 63	Lobulated hyperplastic growth measuring approximately 4 × 1 cm in the left mandibular anterior-to-premolar segment
Radiographic findings	Ghost-tooth appearance with enlarged pulp chambers, short open-apexed roots, and delayed eruption of successors	Ghost-tooth appearance with enlarged pulp spaces, short open-apexed roots, and widened pericoronal radiolucency around tooth 31
Ground section findings	Thin hypoplastic enamel, indistinct DEJ lacking scalloping, extensive interglobular dentin, widened predentin, and enlarged pulpal space	Thin hypoplastic enamel, indistinct DEJ lacking scalloping, extensive interglobular spaces, disorganized dentinal tubules, and enlarged pulpal space
Polarized light findings	Patchy blue-gray birefringence with non-birefringent interglobular zones and polychromatic orange-red and blue interference colors	Heterogeneous patchy birefringence with abnormal orange-red zones, intensely bright DEJ, and weakly birefringent interglobular areas
Decalcified histopathology	Enameloid conglomerates within dentinal matrix, irregular dentinal tubules, pulp stones, and extravasated erythrocytes	Enameloid aggregates within dentinal matrix, wide predentin zones, and extravasated erythrocytes
Associated soft-tissue histopathology	-	Inflamed hyperplastic dental follicle with proliferating odontogenic epithelial rests and CWNs
Diagnosis	Regional odontodysplasia	Regional odontodysplasia with inflamed CHDF
Treatment	Extraction of teeth 61 and 64 following acute abscess; strip composite crowns on teeth 65 and 75	Incisional biopsy, extraction of affected teeth, followed by prosthetic and orthodontic rehabilitation
Follow-up	-	Reviewed at 6 weeks with satisfactory healing; under active follow-up

Limitations

This report describes only two cases, and hence, the findings are not generalizable to ROD as a whole. Characterization was limited to light microscopic techniques; immunohistochemical, ultrastructural, molecular, and quantitative mineralization analyses were not performed and would be necessary for more definitive characterization of the mineralization defect and the composition of the follicular calcifications. No vascular assessment was undertaken, and the proposed etiologic mechanisms remain hypothetical.

## Conclusions

These two cases expand the documented clinical and histopathologic spectrum of ROD. The first case illustrates an uncommon pattern of continuous ipsilateral bimaxillary involvement in the mixed dentition, involving both primary teeth and a permanent molar, documented over five years of serial radiographic observation. The second case demonstrates unilateral mandibular involvement in the permanent dentition, distinguished by the additional finding of an inflamed, calcifying hyperplastic dental follicle harboring enameloid conglomerates. Polarized light microscopy, employed in both cases, demonstrated loss of the uniform birefringence characteristic of normally mineralized dentin, providing an additional structural dimension to the histopathologic documentation. The primary learning point is that in ROD involving unerupted or impacted teeth, histopathologic examination of the associated follicular soft tissue should be considered, particularly when enlarged pericoronal tissue or a widened pericoronal radiolucency is present. The follicle may harbor calcifying hyperplastic changes that radiographically simulate odontogenic cysts or neoplasms and may provide additional diagnostic information for the pathologic characterization of the case.
